# High-Yield Room-Temperature Aryl ^211^At Radiolabeling via Redox-Activated Column-Shipped ^211^At

**DOI:** 10.1021/acsorginorgau.6c00015

**Published:** 2026-06-15

**Authors:** Jehan S. Perera, Jennifer M. Pyles, Adrianna L. Orsi, Christine C. Lawrence, Marcus Le, Gabriel C. Tabacaru, Lauren A. McIntosh, Sherry J. Yennello, Jonathan D. Burns

**Affiliations:** † Department of Chemistry, 9968 University of Alabama at Birmingham, Birmingham, Alabama 35294, United States; ‡ Cyclotron Institute, 14736 Texas A&M University, College Station, Texas 77843, United States; § Department of Chemistry, Texas A&M University, College Station, Texas 77843, United States

**Keywords:** astatine-211, astatodeboronation, ^211^At-aryl labeling, astatine shipment, targeted alpha therapy

## Abstract

Efficient late-stage formation of aryl–^211^At bonds is relevant to expanding the scope of targeted α-therapy radiopharmaceuticals. In this work, a catalyst-enabled astatodeboronation approach is evaluated using wet chemically regenerated ^211^At recovered from shipped 3-octanone-impregnated columns. A series of aryl boronic esters bearing electron-donating and electron-withdrawing substituents were radiolabeled under mild conditions, with reaction parameters examined through time-dependent studies. Radiochemical conversions ranging from 68–97% were observed across the substrate set. Density functional theory calculations were employed as a qualitative framework to contextualize relative B–C and At–C bonding descriptors. Overall, this study demonstrates that wet chemically regenerated ^211^At can be applied to the late-stage labeling of aryl boronic esters under mild conditions and provides guidance for future exploration of ^211^At radiolabeling strategies.

## Graphical abstract

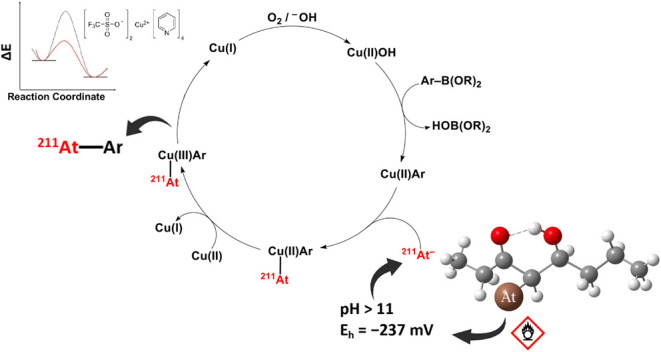

## 1 Introduction

Targeted Alpha Therapy (TAT) is an emerging modality of precision medicine, which provides a high level of cytotoxicity over a short-range due to the high linear energy transfer (LET) of the emitted α-particle.^1^ The range of the α-particle is on the order of ∼50 μm, which provides a lethal dose of radiation to neighboring cells within a diameter of ∼100 μm, and is closely matched to microscopic tumor dimensions,^2,3^ while the relatively short-range limits collateral injury to normal tissue.^4^ Several α-emitters have been assessed for TAT applications, including^149^Tb, ^211^At, ^212^Bi (from ^212^Pb), ^213^Bi, ^223^Ra, ^225^Ac, ^226^Th, ^227^Th, and ^230^U, and many of them have reached clinical or advanced preclinical evaluation.^5^ Among these, ^211^At is one of the most promising due to its convenient half-life, ∼ 7.2 h, and elegant decay scheme, resulting in a quantitative α-emission per decay.^6^ However, routine access to ^211^At remains challenging.^7^ The predominant production route, ^209^Bi­(α,2n)^211^At on medium-energy cyclotrons,^8–11^ demands careful targetry and postirradiation handling, while ^211^Rn→^211^At generator technology^12^ is still maturing, and it is unclear whether it will become a viable option. The 7.21-h half-life imposes stringent logistical and radiosynthetic constraints, favoring shipment modalities that minimize handling and enable immediate downstream use.^7^

Although direct speciation of ^211^At has not been achieved due to the limits of its production (pmol quantities),^13^ the reactive At species can be inferred through the control of redox potential and pH. There are three oxidation states believed to be stable in aqueous systems, At­(−I), At­(+I), and At­(+III), and a proposed Pourbaix diagram has been generated.^13,14^ This framework reconciles the apparent ambiguity surrounding At’s electrophilic or nucleophilic behavior across different reaction systems. Extraction studies further demonstrate that counterions, such as nitrate, can influence At speciation and coordination by modifying the local electronic environment, rather than acting as passive spectators, thereby affecting partitioning behavior and ligand interactions in nitric acid media.^15,16^ Complementary investigations involving carbonyl-containing ligands suggest that partial covalent character may contribute to the stabilization of specific coordination motifs, providing molecular-level insight into factors governing At reactivity and extraction at tracer concentrations.^17–21^

Current ^211^At labeling strategies encompass three principal approaches: astatodestannylation via electrophilic ipso-substitution of organotin precursors, the most extensively validated method across diverse molecular scaffolds;^22^ diaryliodonium-based nucleophilic astatination, which circumvents organotin toxicity under mild conditions;^23^ and prosthetic group conjugation for biologics incompatible with direct astatination.^2^ In this context, copper-mediated astatodeboronation of arylboronic acids and esters has emerged as a particularly attractive option, offering reduced toxicity, improved functional-group tolerance, and simplified purification.^24^ Building on copper-mediated radioiodination chemistry, Reilly et al. demonstrated rapid astatination of aryl and heteroaryl boronic esters within 10 min at room temperature, enabling late-stage formation of aryl–^211^At bonds under mild conditions.^24^ Berdal et al. demonstrated efficient, water-based one-step ^211^At labeling of arylboronic acids, achieving quantitative RCY for a model substrate and >95% RCY for antibody conjugation under mild conditions.^25^ In these reactions, the active labeling agent is best described as nucleophilic ^211^At, requiring a reduced form of ^211^At and the necessary conditions to stabilize the desired species.^14,26^ Recent reviews^13,27^ highlight both its promise and persistent challenges in yield consistency, substrate generality, and mechanistic clarity, while underscoring the intrinsic predisposition of aryl boron precursors toward electrophilic ipso substitution.

The limited availability and rapid decay of ^211^At, combined with the difficulty of experimentally resolving solution speciation at tracer concentrations, make theoretical modeling an essential tool for interpreting radiolabeling behavior and guiding reaction design. Hybrid dispersion-corrected DFT methods, such as B3LYP-D3­(BJ), which combine Becke’s three-parameter hybrid exchange with Lee–Yang–Parr correlation and Grimme’s D3­(BJ) dispersion correction,^28^ have been applied to organometallic and At-containing molecular models and shown to provide qualitatively consistent descriptions when paired with flexible triple-ζ basis sets (e.g., def2-TZVPP)^29^ for describing aryl C–B frameworks and the formation of C–At bonds; the performance of DFT methods for At-containing systems has been specifically benchmarked.^28^ Early At computational studies relied on COSMO and IEFPCM continuum solvation models, which, despite reasonable accuracy in reproducing redox trends, require explicit cavity definitions that introduce user-dependent variability-a particular concern for At given its high polarizability.^26,30^ The SMD model addresses this limitation through empirically parametrized atomic radii, reducing user input and improving reproducibility.^31^ Electronic descriptors, including Wiberg bond indices (WBI),^32^ atomic charges, and Natural Bond Orbital (NBO)^33^ donor–acceptor interactions, are used here as qualitative descriptors of electronic structure rather than definitive bonding metrics.

This study examines the radiolabeling of aryl boronic esters using ^211^At recovered from shipped column cartridges via a previously validated wet chemical regeneration protocol.^34^ A single-step preconditioning recovers reactive ^211^At at near-quantitative levels, which is then directly coupled to copper-mediated astatodeboronation under mild conditions (methanol, room temperature, minutes). The reactivity of the recovered ^211^At with a variety of aryl boronic esters containing both EDGs and EWGs substituents is of particular interest, providing a simple and operationally robust platform that can be readily implemented using standard wet chemical techniques at both production and recipient sites.

## 2 Results and Discussion

The reactivity of ^211^At recovered from the shipped column cartridges was analyzed using a series of aryl boronic ester precursors (Figure 1), where the substrates were varied by adding EDGs and EWGs to the aryl system to demonstrate that this approach could be used for a wide variety of substrates.^35^ The conditions necessary for copper-mediated C–At bond formation to proceed (pH ≈ 11.8, E_h_ ≈ −237 mV)^20,24^ should result in the reduced At^–^ species (Scheme 1).^13^ The labeling reaction was expected to proceed with all six substrates, through copper-mediated activation and subsequent C–At bond formation.

**Figure 1:**
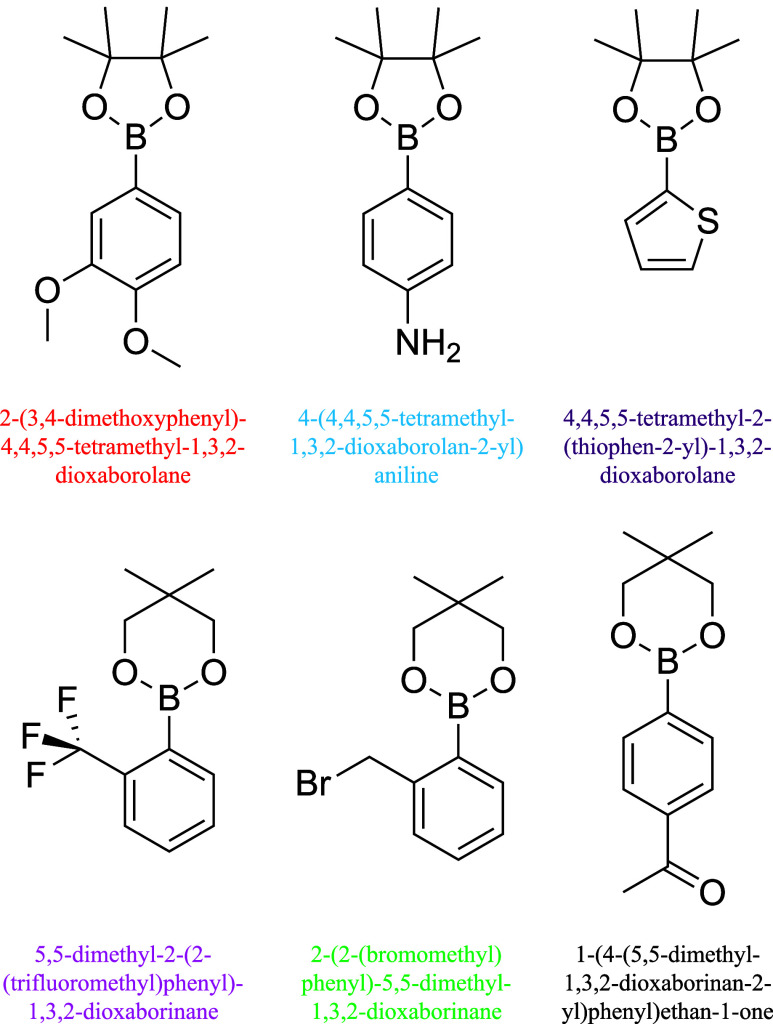
Electron-donating (top row) and electron-withdrawing (bottom row) aryl boronates used for copper-mediated radiolabeling with ^211^At.

**Scheme 1:**
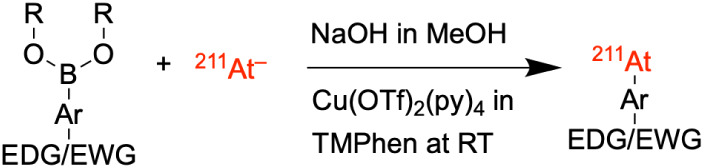
Copper-Mediated Astatodeboronation of EDG/EWG-Substituted (hetero)­Aryl Boronic Esters under Standard Conditions: Reactive Aqueous ^211^At Species Under NaOH/Methanol Conditions, [Cu­(OTf)_2_(py)_4_], and TMPhen at RT

### 2.1 Electron-Donating Aryl Boronates Used for Copper-Mediated ^211^At Labeling

The astatodeboronation (Scheme 1) of 2-(3,4-dimethoxyphenyl)-4,4,5,5-tetramethyl-1,3,2-dioxaborolane was examined first as an EDG benchmark. This donor-activated aryl boronic ester contains *meta* and *para* methoxy substituents, which are associated with increased aryl π-electron density while introducing minimal *ortho* steric congestion at the reactive ipso position. As shown in Figure 2, the RCC (radiochemical conversion) increased as a function of reaction time, reaching ∼75% at 5 min and ∼83% at 30 min. The labeling efficiency for the methoxy derivative was higher than that observed for the aniline derivative, which will be discussed in detail later, and is believed to be due to the dual methoxy groups, which provide potential n­(O)→Cu interactions that preorganize the substrate-catalyst complex.^36^

**Figure 2:**
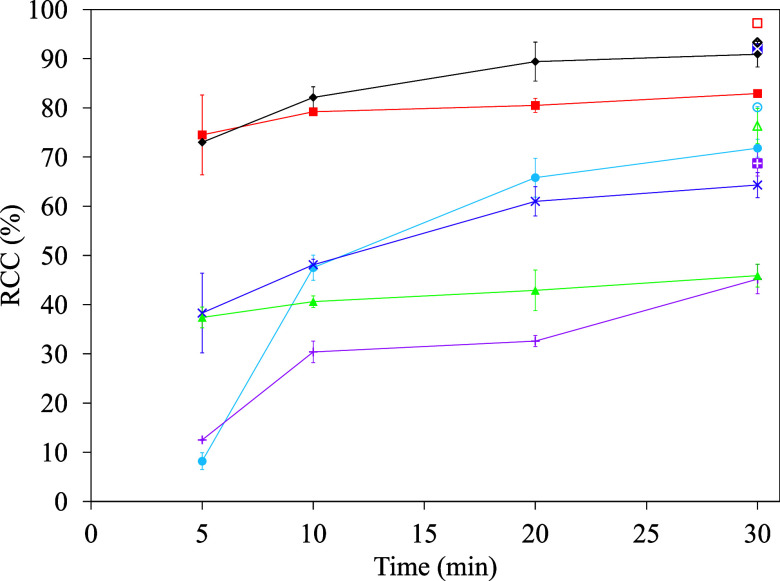
RCC for the astatodeboronation as a function of time for 2-(3,4-dimethoxyphenyl)-4,4,5,5-tetramethyl-1,3,2-dioxaborolane (■), 4-(4,4,5,5-tetramethyl-1,3,2-dioxaborolan-2-yl)­aniline (●), 4,4,5,5-tetramethyl-2-(thiophen-2-yl)-1,3,2-dioxaborolane (×), 5,5-dimethyl-2-(2-(trifluoromethyl)­phenyl)-1,3,2-dioxaborinane (+), 2-(2-(bromomethyl)­phenyl)-5,5-dimethyl-1,3,2-dioxaborinane (▲), and 1-(4-(5,5-dimethyl-1,3,2-dioxaborinan-2-yl)­phenyl)­ethan-1-one (◆), with hollow symbols at 30 min representing a doubling of the Cu­(OTf)­2­(py)­4 and TMPhen concentration. Lines are drawn to guide the eye. Color coding matches Figure 1.

Doubling both Cu­(OTf)_2_(py)_4_ and TMPhen concentrations was associated with increased RCC to ∼97 ± 0.11%, consistent with improved labeling efficiency under copper and ligand-rich conditions. As will be discussed in a later section, this is believed to be caused by a Cu­(II)–Cu­(II) redox reaction to form Cu­(III) in the catalyst, allowing reductive elimination to proceed, releasing the ^211^At-labeled compound.^36^ In closely related Cu-mediated astatodeboronation studies, Reilly et al.^24^ reported astatination of aryl pinacol boronic esters, with RCC spanning approximately 70% to >90%. Although the substrates examined here differ from Reilly et al., the observed sensitivity to Cu and ligand loading is consistent with these prior findings. The high RCC obtained under ambient conditions underscores the robustness of the labeling protocol and indicates that redox-recovered ^211^At from the shipped column cartridges is amenable to these types of labeling reactions.^20,34^

Next, building on our earlier work with this 4-(4,4,5,5-tetramethyl-1,3,2-dioxaborolan-2-yl)­aniline, where 36 ± 22% ^211^At labeling was observed after 10 min^20^, current reaction conditions were implemented to achieve a labeling efficiency of roughly 72% after 30 min (Figure 2). In comparison with the methoxy derivative, these results reveal behavior that deviates from simple Hammett-based expectations. The aniline substrate, bearing a single powerful electron donor (*para*-NH_2_, σ_p_ = −0.66), might be expected to outperform the dimethoxyphenyl analog, which possesses two weaker methoxy groups with cumulative electron donation of only Σ_σ_ ≈ −0.15 (σ_p_ = −0.27 for *para*-OCH_3_; σ_m_ = +0.12 for *meta*-OCH_3_).^35^ However, the observed RCC followed the opposite trend, with a 72% RCC compared to an 83% RCC. This inversion further supports that the methoxy pendants are participating in the coordination of the Cu metal center and enhancing the catalytic effect.

Again, increases in the labeling efficiency were observed with increased catalyst loading, reaching approximately 80% upon doubling the catalyst concentration at 30 min. This reaction demonstrates that *p*-amino substituents, critical for bioconjugation and peptide radiolabeling, can be incorporated into ^211^At-labeled compounds.

To move beyond aryl boronic esters bearing exocyclic heteroatom substituents (e.g., methoxy or amino groups), we investigated 4,4,5,5-tetramethyl-2-(thiophen-2-yl)-1,3,2-dioxaborolane as a representative sulfur heterocycle. The labeling efficiency rose from about 38% at 5 min to ∼65% at 30 min. Increasing the catalyst and ligand loadings resulted in a substantial enhancement, with the labeling efficiency reaching ∼92% at 30 min, corresponding to an approximately 1.7-fold improvement and representing the highest sensitivity to catalyst loading observed across the substrate set (Figure 2). This optimized yield closely matches the RCC reported by Reilly et al.^24^ for thiophene using freshly prepared^211^At within 10 min, yielding comparable performance (∼90%). These results demonstrate that redox-recovered ^211^At from the shipped column cartridges can be utilized for labeling heteroaromatic substrates. Such behavior may reflect sulfur-mediated preorganization of the catalytic environment to enhance local reactivity at the heteroaromatic ring, similar to what was observed by Reilly et al. with nitrogen groups.^24^

### 2.2 Electron-Withdrawing Aryl Boronates Used for Labeling with ^211^At

Electron-withdrawing substituents on aromatic frameworks were then evaluated to determine their impact on labeling efficiency and apparent reactivity in boronic ester with ^211^At, including acyl, −CH_2_Br, and −CF_3_ substituents (Figure 1). A key feature of the CF_3_-substituted aryl boronate is the pronounced attenuation in RCC relative to donor-activated analogues. Whereas electron-rich aryl boronates in this study routinely achieve RCC exceeding 85–90% within 30 min, the *ortho*- CF_3_ derivative afforded a meager 45% and reached only ∼ 69% upon doubling the Cu­(OTf)_2_(py)_4_ and TMPhen concentrations (Figure 2). Notably, these values remain in line with those typically reported for copper-mediated radiofluorination, 60–75%.^37^

The benzylic bromomethyl substituent aryl boronate displayed RCC that exceeds that of the *ortho*-CF_3_ analogue at early reaction times, ∼37% at 5 min, and the efficiency at 30 min is indistinguishable, ∼ 46%. Upon doubling the catalyst concentration, the reaction reached roughly 76% at 30 min (Figure 2). These results are promising compared with iododeboronation reactions on analogous substrates, which typically require more forcing conditions to attain comparable yields, consistent with the distinct mechanistic pathways operative in C–At versus C–I bond formation from arylboronate precursors.^24,38,39^

Following the diminished reactivity observed for ortho-substituted aryl boronates, the para-acyl-substituted substrate exhibits a pronounced recovery in labeling efficiency, rising rapidly to >80% within the first 10 min and subsequently reaching a high-yield plateau of ∼93% at 30 min (Figure 2). Increasing the catalyst concentration provided no improvement in labeling efficiency, indicating that catalyst availability is not rate-limiting under these conditions, consistent with a system operating in an optimized regime in which the efficiency is already near-maximal, as demonstrated for Cu­(OTf)_2_ cocatalysis by Wang and Stahl.^40^ This suggests that the observed yield plateau reflects proximity to a thermodynamic ceiling rather than a kinetic constraint.

### 2.3 Theoretical Insights Underpinning Experimental Reactivity

To try to understand the labeling reactivity, a series of DFT calculations were performed, including NBO and WBI analyses on the boronic esters and their astatinated products (Figures 3 and 4). Additionally, although the formation of the aryl-astatine bond is accompanied by an increase in the C–At bond length relative to the precursor B–C_*ipso*_ linkage, the corresponding WBI values increase substantially (from 0.82–0.91 for B–C_*ipso*_ to 0.98–1.12 for At–C_*ipso*_), indicating a pronounced increase in covalent character upon astatination. This decoupling of bond length and bond order is characteristic of heavy-element bonding and is consistent with the established relationship between WBI and bond strength, underscoring the electronic stabilization of the C–At bond in the radiolabeled products.^32^

**Figure 3:**
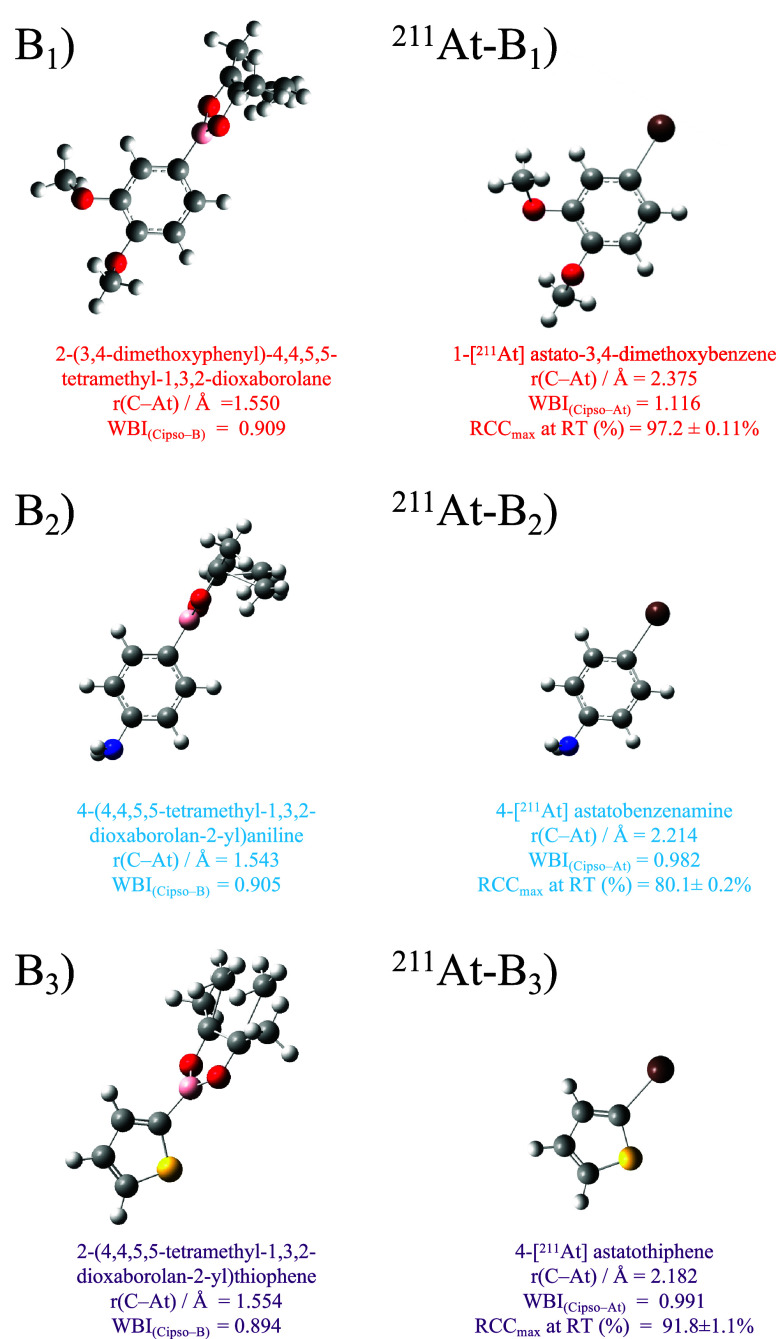
EDG-substituted aryl boronic esters (B_1_–B_3_) and their [^211^At] aryl products (At–B_1_ – At–B_3_). For each structure, r­(C–At bond length), WBI, and RC*C*_max_ are reported. Color coding matches Figure 1.

**Figure 4:**
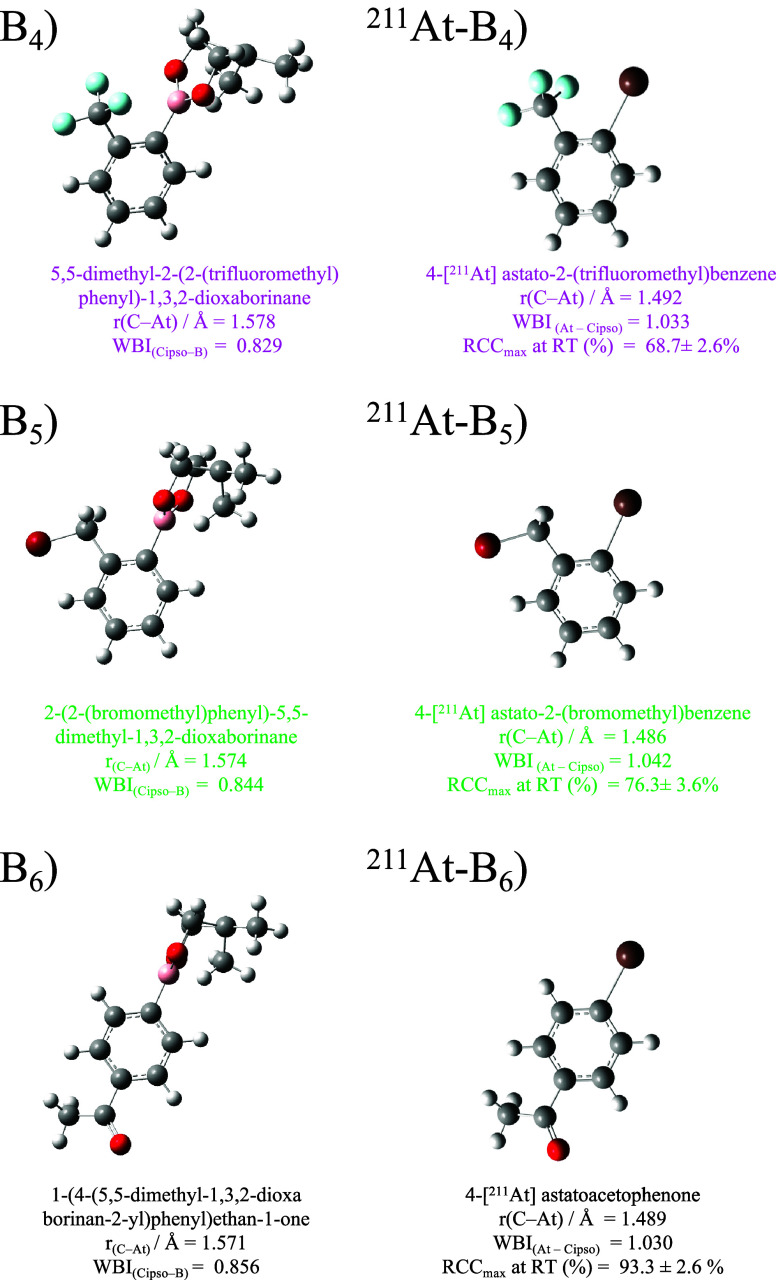
EWG-substituted aryl boronic esters (B_4_–B_6_) and their [^211^At] aryl products (At–B_4_ – At–B_6_). For each structure, r­(C–At), WBI, and RC*C*_max_ are reported. Color coding matches Figure 1.

The qualitative invariance of NBO and WBI descriptors across four independent computational protocols (Tables S1, S2, S3, and S4) further supports the reliability of this approach for comparative descriptor analysis. Sergentu et al.^28^ demonstrated that B3LYP performs well for organoastatine geometries and electronic properties under scalar-relativistic ECP treatment, establishing it as a reliable functional for At-descriptor analysis. Galland et al.^23,41^ further showed that qualitative trends in astatine bonding descriptors remain consistent under scalar-relativistic treatment. Guo et al.^42^ additionally demonstrated that scalar-relativistic DFT successfully rationalizes experimental astatine bonding trends in organoastatine compounds. Accordingly, the present NBO and WBI descriptors are interpreted as qualitative comparative indicators, and quantitative mechanistic conclusions are deliberately avoided.

### 2.4 Proposed Mechanism for Catalyzed Astatodeboronation

Guided by the proposed astatine Pourbaix diagram,^13^ our reaction conditions (pH ∼ 11.8, E_h_ ∼ −237 mV) place the system within a regime where reduced At^–^ species are thermodynamically accessible; within this context, the catalytic cycle proposed in Scheme 2 provides a useful framework for interpreting the observed trends across the aryl boronate series.^26^ In particular, the first step involving aryl engagement with the copper metal center, most plausibly during aryl transfer from B to Cu­(II), is expected to occur at the B–C_*ipso*_ bond. Notably, the high efficiency of the thiophene, para-acetyl ketone, and 3,4-dimethoxyphenyl derivatives, which exhibit RCCs of ∼92%, ∼93%, and ∼97%, respectively, points to an additional mechanistic contribution, namely the coordination of the S or O atoms to the Cu­(II) metal center. These interactions would preorganize the substrate-catalyst complex and enhance the catalytic activity.^36^ This is a well-known aspect of copper-catalyzed reactions, and has been observed in carbonyl-containing boronic esters, which undergo efficient transformations due to weak coordination with the Cu metal center.^43,44^

**Scheme 2:**
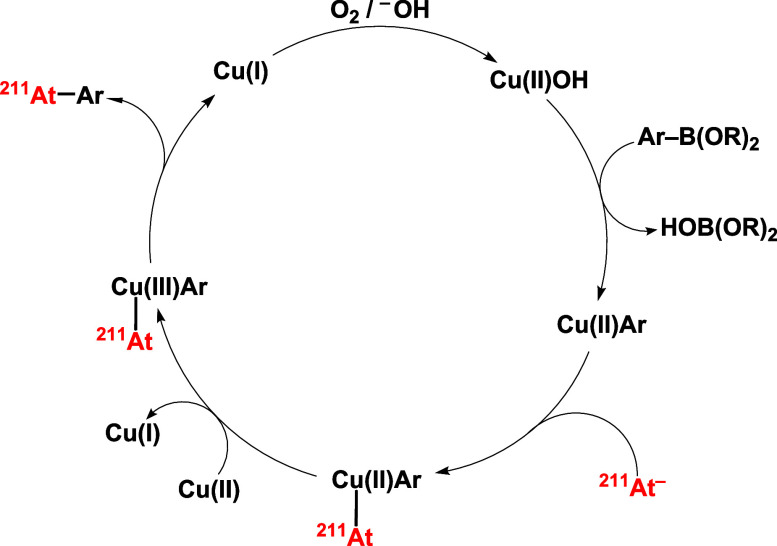
Proposed Catalytic Cycle for Cu-Mediated Astatodeboronation of Aryl Boronates to ^211^At–Aryl

Increasing the concentration of Cu­(OTf)_2_(py)_4_ and TMPhen enhanced the RCC for all six derivatives studied, suggesting that higher Cu­(II) concentration enhances the oxidation of the catalytic Cu­(II) metal center to Cu­(III), an otherwise disfavored step in the cycle. Notably, iododeboronation systems exhibit a broader electronic tolerance window, consistent with the lower energetic penalty associated with C–I bond formation relative to C–At coupling, providing a mechanistic basis for the heightened substituent sensitivity observed here.^45^

## 3 Experimental Section

### 3.1 Materials

Methanol (ACS grade, ≥ 99.8%, CH_3_OH) and ethyl acetate (ACS grade, ≥ 99.5%, C_4_H_8_O_2_) were purchased from VWR; sodium hydroxide (ACS grade, ≥ 98%, NaOH) was purchased from Fisher Scientific; 3,4,7,8-tetramethyl-1,10-phenanthroline (analytical grade, ≥ 98%, TMPhen) was purchased from BTC; tetrakis­(pyridine)­copper­(II) triflate (ACS grade, 98.75%, C_22_H_20_CuF_6_N_4_O_6_S_2_), 2-(2-(bromomethyl)­phenyl)-5,5-dimethyl-1,3,2-dioxaborinane (analytical grade, ≥ 95%, C_12_H_16_BBrO_2_), 4-(4,4,5,5-tetramethyl-1,3,2-dioxaborolan-2-yl)­aniline (ACS grade, ≥ 98%, C_12_H_18_BNO_2_), 1-(4-(5,5-dimethyl-1,3,2-dioxaborinan-2-yl)­phenyl)­ethan-1-one (analytical grade, ≥ 98%, C_13_H_17_BO_3_), 5,5-dimethyl-2-(2-(trifluoromethyl)­phenyl)-1,3,2-dioxaborinane (ACS grade, ≥ 98%, C_12_H_14_BF_3_O_2_), and 2-(3,4-dimethoxyphenyl)-4,4,5,5-tetramethyl-1,3,2-dioxaborolane (analytical grade, ≥ 95%, C_14_H_21_BO_4_) were purchased from Ambeed; sodium hypochlorite solution (6–14% active chlorine, NaOCl) was purchased from Sigma–Aldrich; and 2-(4,4,5,5-tetramethyl-1,3,2-dioxaborolan-2-yl)­thiophene (ACS grade, ≥ 98%, C_10_H_15_BO_2_S) was purchased from MilliporeSigma; and all were used as received. Deionized (D.I) H_2_O was obtained from an ELGA LabWater Purelab Flex ultrapure laboratory water purification system operated at 18.2 MΩ·cm at 25 °C.

### 3.2 Methods

Quantitative analysis for ^211^At was performed via gamma (γ)-ray spectroscopy using a calibrated ORTEC GEM Coaxial P-type HPGe Gamma-Ray Detector (HPGe) with a relative efficiency of 20.0% and an active detector volume of ∼115 cm^3^ and DSPEC-50 digital signal analyzer along with MAESTRO software. The detector has an energy resolution of 0.82 at 122 keV and 1.33 at 1330 keV. Relevant nuclear data were obtained from Browne and Firestone.^46^ All calibrations were determined with a ^152^Eu and a ^207^Bi standard γ-ray source, both traceable to the National Institute of Standards and Technology (NIST), purchased from Eckert & Ziegler Isotope Products. ^211^At was tracked directly using the 76.9, 79.3, 89.8, and 92.3 keV X-rays and the 687 keV γ-ray. All experiments were performed in triplicate. WARNING: ^211^At is highly radioactive and was handled under ALARA principles in laboratories equipped to handle radioactive materials appropriately.

### 3.3 Shipment and Recovery Of ^211^At from 3-Octanone–impregnated Columns

The ^211^At was shipped from Texas A&M University to the University of Alabama at Birmingham in a 1.0 mL Thermo Scientific Pierce Spin Column packed with 0.5 mL of 3-octanone-impregnated Amberchrom CG300 M porous beads, which have a pore volume of 0.7 mLg^–1^ and a particle size of 50–100 μm, as described in detail in our previous work.^17,20,34,47^ The columns were conditioned with 2 bed volumes (BV) of H_2_O and the ^211^At was eluted from the column using fresh 3-octanone in several fractions.^19,34,47,48^ The column, before and after ^211^At recovery, along with conditioning and elution fractions, was analyzed using the HPGe to follow the ^211^At recovery. All 3-octanone elution fractions containing ^211^At were combined and contacted with 1.0 mL of 1.5 M NaOCl, then agitated by end-overend rotation (≈18 rpm) for 10 min at room temperature to promote oxidative cleavage of C–At bonds and centrifuged for 5 min to ensure complete phase separation.^34^ The majority of the aqueous phase (0.80 mL) was withdrawn, and contained the bulk of the ^211^At as what is believed to be an oxidized species. The residual organic phase was returned to the vessel, charged again with a fresh 1.0 mL portion of 1.5 M NaOCl, and the extraction sequence was repeated multiple times, recovering ^211^At into the aqueous phase at each cycle. The combined aqueous fractions were diluted with deionized water to the target ^211^At concentration for subsequent labeling experiments.

### 3.4 Protocol for Deborylative Astatination

At room temperature, each aryl boronic ester (2-(2-(bromomethyl)­phenyl)-5,5-dimethyl-1,3,2-dioxaborinane; 4-(4,4,5,5-tetramethyl-1,3,2-dioxaborolan-2-yl)­aniline; 1-(4-(5,5-dimethyl-1,3,2-dioxaborinan-2-yl)­phenyl)­ethan-1-one; 5,5-dimethyl-2-(2-(trifluoromethyl)­phenyl)-1,3,2-dioxaborinane; 2-(4,4,5,5-tetramethyl-1,3,2-dioxaborolan-2-yl)­thiophene; or 2-(3,4-dimethoxyphenyl)-4,4,5,5-tetramethyl-1,3,2-dioxaborolane) was dissolved or diluted individually in anhydrous methanol. With gentle swirling, 0.10 M NaOH in methanol (100 μL) was added dropwise, followed by the oxidized astatine solution (100 μL, ∼250 kBq of ^211^At); the mixture was briefly swirled to ensure homogeneity. An aqueous stock of 20 mM or 40 mM [Cu­(OTf)_2_(py)_4_] in methanol (100 μL) was then added, followed by a methanol stock of 30 mM or 60 mM TMPhen (100 μL). All reactions were performed in triplicate in sealed vials and agitated by end-overend rotation (∼18 rpm) until the specified time points; at each time point, mixtures were centrifuged for 5 min, and aliquots were withdrawn and applied to TLC plates for radio-TLC analysis.

### 3.5 Radio-Thin Layer Chromatography (Radio-TLC)

Thin layer chromatography (TLC) was carried out using a Silica gel 60 TLC aluminum plate purchased from Sigma–Aldrich. The plates were with a 6-μL aliquot and eluted for 80 mm with ethyl acetate. After elution, the plates were analyzed using an AR-2000 radio-TLC imaging scanner (Eckert & Ziegler Radiopharma, Inc.). The data were obtained using WinScan3 software. All experiments were performed in triplicate. The radiochemical conversion (RCC) was determined by dividing the area of each band by the total number of counts on each plate and is reported as mean ± SD of the triplicates. Uncertainties in R_f_ reflect the full width at half-maximum (FWHM) of each band.

### 3.6 Computational Methods

Details on the computational methods are located in the Supporting Information.

## 4 Conclusions

In conclusion, ^211^At recovered from shipped column cartridges has been shown to have high reactivity toward copper-mediated astatodeboronation of aryl groups. This is facilitated by a simple wet chemical redox regeneration of ^211^At from 3-octanone shipping columns. The reactivity was tested across several model compounds, ranging from electron-donating, where high RCCs were observed (80–97%), to electron-withdrawing groups, with slightly lower RCCs (68–93%). Varying the catalyst concentration resulted in an equally large impact on the RCC. From these results, it seems weak coordination of the Cu­(II) metal center promotes higher RCC, by presumably through preorganization of the complex to lower the activation energy of the aryl–B bond cleavage and formation of the aryl-Cu­(II). Ongoing and future studies expanding substrate scope and integrating complementary experimental and computational probes are needed to better understand any relationship between structure and reactivity, and will be the focus of future studies. Even in cases where the precursor exhibits less-than-ideal characteristics for labeling, the RCCs are still competitively viable compared to other labeling routes and show promise in further investigations into using this approach for radiopharmaceutical development in the quickly expanding field of TAT.

## Supplementary Material



## Data Availability

The data underlying this study are available in the published article and its Supporting Information.

## Notes

The document presents supporting data for rapid room-temperature aryl-^211^At radiolabeling via copper-mediated astatodeboronation, including time-resolved radio-TLC results, computational analyses, and optimized molecular coordinates (DOCX)
