# Supplementary material for: High-Yield Room-Temperature Aryl 211At Radiolabeling via Redox-Activated Column-Shipped 211At
Source: ACS Org Inorg Au. 2026 Jun 15;6(4):414–21. doi: 10.1021/acsorginorgau.6c00015 (PMC13449431; doi:10.1021/acsorginorgau.6c00015)
Supplement: Supplementary file 1 [file gg6c00015_si_001.docx]

**High-Yield Room-Temperature Aryl ^211^At Radiolabeling via Redox-Activated** **Column-Shipped ^211^At.**

Jehan S. Perera^1^, Jennifer M. Pyles^1^, Adrianna L. Orsi^1^, Christine C. Lawrence^2^, Marcus Le^2^, Gabriel C. Tabacaru^2^, Lauren A. McIntosh^2^, Sherry J. Yennello^2,3^, and Jonathan D. Burns^*,1^

^1^Department of Chemistry, University of Alabama at Birmingham, Birmingham, AL 35294, USA

^2^Cyclotron Institute, Texas A&M University, College Station, TX 77843, USA

^3^Department of Chemistry, Texas A&M University, College Station, TX 77843, USA

For submission to publish in the

*ACS Organic & Inorganic Au*

as a full-length article.

This submitted report has been authored by the University of Alabama at Birmingham, under Award No. DE-SC0024600 with the U.S. Department of Energy. The United States Government retains and the publisher, by accepting the article for publication, acknowledges that the United States Government retains a non-exclusive, paid-up, irrevocable, world-wide license to publish or reproduce the published form of this manuscript, or allow others to do so, for the United States Government purposes. *The Department of Energy will provide public access to these results of federally sponsored research in accordance with the DOE Public Access Plan (*[*http://energy.gov/downloads/doe-public-access-plan*](http://energy.gov/downloads/doe-public-access-plan)*).*

This report was prepared as an account of work sponsored by an agency of the United States Government. Neither the United States Government nor any agency thereof, nor any of their employees, makes any warranty, express or implied, or assumes any legal liability or responsibility for the accuracy, completeness, or usefulness of any information, apparatus, product, or process disclosed, or represents that its use would not infringe privately owned rights. Reference herein to any specific commercial product, process, or service by trade name, trademark, manufacturer, or otherwise does not necessarily constitute or imply its endorsement, recommendation, or favoring by the United States Government or any agency thereof. The views and opinions of authors expressed herein do not necessarily state or reflect those of the United States Government or any agency thereof.

Research supported by U.S. Department of Energy Office of Isotope R&D and Production and the DOE Established Program to Stimulate Competitive Research (EPSCoR) under Award No. DE-SC0024600. CCL, ML, GCT, LAM, and SJY acknowledge financial support from U.S. Department of Energy Isotope Program, managed by the Office of Science for Isotope R&D and Production under Award No. DE-SC0020958, and Texas A&M University through the Bright Chair in Nuclear Science. Additionally, this work was enabled by the Texas A&M Nuclear Solutions Institute and U.S. Department of Energy under Award No. DE-FG02-93ER40773.

**Supporting Information**

**High-Yield Room-Temperature Aryl ^211^At Radiolabeling via Redox-Activated** **Column-Shipped ^211^At.**

Jehan S. Perera^1^, Jennifer M. Pyles^1^, Adrianna L. Orsi^1^, Christine C. Lawrence^2^, Marcus Le^2^, Gabriel C. Tabacaru^2^, Lauren A. McIntosh^2^, Sherry J. Yennello^2,3^, and Jonathan D. Burns^*,1^

^1^Department of Chemistry, University of Alabama at Birmingham, Birmingham, AL 35294, USA

^2^Cyclotron Institute, Texas A&M University, College Station, TX 77843, USA

^3^Department of Chemistry, Texas A&M University, College Station, TX 77843, USA

*e-mail: burnsjon@uab.edu

**Experimental Section**

*Computational Methods:*

DFT calculations were performed using Gaussian 16 (Revision C.01) to examine the electronic structure of aryl boronic ester precursors and their corresponding astatinated products. Geometries were optimized at the B3LYP-D3(BJ) level of theory^1, 2^ with a mixed basis-set strategy: def2-SVP for H, C, N, O, B, F, and S, and def2-TZVPP with associated relativistic effective core potentials for astatine.^3^ Solvation effects were included using the SMD continuum model with methanol as solvent.^4^ To ensure consistency in heavy-element cavity construction, the astatine solvation radius was explicitly specified using SCRF=read and held constant across all calculations.

SCF convergence was facilitated using quadratic convergence (SCF=XQC) when necessary, and geometry optimizations were performed in Cartesian coordinates using the GDIIS algorithm. All optimized structures were verified as true minima by the absence of imaginary frequencies. Electronic structure descriptors were obtained using NBO 3.1 analysis;^5^ Wiberg bond indices (WBI) and optimized bond distances were used to characterize bond order and bond covalency, respectively.^6, 7^ Spin-orbit coupling effects were not explicitly included in the NBO analysis and are acknowledged as a methodological limitation. Method sensitivity was assessed using representative structures by re-optimizing geometries with dispersion corrected B97-D3(BJ) functionals under identical solvation conditions and by reevaluating solvation effects using PCM-family model (IEFPCM) in methanol.^8, 9^ For PCM benchmarking, the UAHF cavity model was employed with a fixed astatine radius of 2.41 Å, consistent with prior studies on astatine redox and speciation.^10^ All reported descriptors correspond to the lowest energy conformer identified for each system. Full benchmarking results, optimized coordinates, and descriptor sensitivity analyses are provided below.

*Time-resolved radio-TLC analysis of EDG- and EWG-substituted boronic esters*

Figure S1: Time-resolved radio-TLC for astatodeboronation of 2-(3,4-dimethoxyphenyl)-4,4,5,5-tetramethyl-1,3,2-dioxaborolane. Product R_f_ = 0.82 ± 0.05. Pre (free oxidized ^211^At) Average R_f_ = 0.00 ± 0.03. The shaded region represents where the area was determined for each band.

Figure S2: Time-resolved radio-TLC for astatodeboronation of 4-(4,4,5,5-tetramethyl-1,3,2-dioxaborolan-2-yl)aniline. Product R_f_ = 0.87 ± 0.03. Pre (free oxidized ^211^At) Average R_f_ = 0.00 ± 0.03. The shaded region represents where the area was determined for each band.

Figure S3: Time-resolved radio-TLC for astatodeboronation of 4,4,5,5-tetramethyl-2-(thiophen-2-yl)-1,3,2-dioxaborolane. Product Average R_f_ = 0.91 ± 0.04. Pre (free oxidized ^211^At) R_f_ = 0.00 ± 0.03. The shaded region represents where the area was determined for each band.

Figure S4: Time-resolved radio-TLC for astatodeboronation of 5,5-dimethyl-2-(2-(trifluoromethyl)phenyl)-1,3,2-dioxaborinane. Product R_f_ = 0.86 ± 0.05. Pre (free oxidized ^211^At) R_f_ = 0.00 ± 0.03. The shaded region represents where the area was determined for each band.

Figure S5: Time-resolved radio-TLC for astatodeboronation of 2-(2-(bromomethyl)phenyl)-5,5-dimethyl-1,3,2-dioxaborinane. Product Average R_f_ = 0.78 ± 0.04. Pre (free oxidized ^211^At) R_f_ = 0.00 ± 0.03. The shaded region represents where the area was determined for each band.

Figure S6: Time-resolved radio-TLC for astatodeboronation of 1-(4-(5,5-dimethyl-1,3,2-dioxaborinan-2-yl)phenyl)ethan-1-one. Product R_f_ = 0.86 ± 0.05. Pre (free oxidized ^211^At) R_f_ = 0.00 ± 0.03. The shaded region represents where the area was determined for each band.

Benchmarking analyses were conducted to evaluate the robustness of the calculated electronic descriptors with respect to functional choice and solvation treatment. Geometry optimizations were performed without subsequent vibrational frequency calculations, as thermochemical parameters were not required for the present study. Structures were treated as stationary points based on satisfying the standard Gaussian 16 (G16) optimization convergence criteria. Unless otherwise noted, all energies reported here are absolute electronic energies in Hartree. WBI denotes the Wiberg bond index, and N_val_ denotes the natural valence electron population at the ipso carbon. The NBO-derived descriptors and absolute electronic energies are reported for the arylboronic ester precursors and the corresponding astatinated products.

Table S1 Computed NBO parameters and absolute electronic energies at the B3LYP-D3BJ/genecp level with the SMD implicit solvation model for water.

| Compound | WBI (C_ipso_–B) | WBI (C_ipso_–At) | Absolute electronic energy of pre-labeling precursor | Absolute electronic energy of post-labeling product |
| --- | --- | --- | --- | --- |
| B1 | 0.9094 | 1.1162 | −872.4016 | −938.1780 |
| B2 | 0.9049 | 0.982 | −697.8724 | −549.4117 |
| B3 | 0.8943 | 0.9917 | −963.2085 | −814.7794 |
| B4 | 0.829 | 1.033 | −940.4009 | −1045.5106 |
| B5 | 0.8435 | 1.0422 | −3216.1270 | −3321.2899 |
| B6 | 0.856 | 1.0304 | −755.7928 | −861.2664 |

Table S2 Computed NBO parameters and absolute electronic energies at the B3LYP-D3BJ/genecp level with the IEFPCM implicit solvation model for water.

| Compound | WBI (C_ipso_–B) | WBI (C_ipso_–At) | Absolute electronic energy of pre-labeling precursor | Absolute electronic energy of post-labeling product |
| --- | --- | --- | --- | --- |
| B1 | 0.9112 | 1.111 | -872.3991 | -938.1734 |
| B2 | 0.9029 | 0.9836 | -963.1051 | -549.4103 |
| B3 | 0.9589 | 0.9928 | −963.1051 | -814.7804 |
| B4 | 0.8285 | 1.0265 | -940.401 | -1045.5101 |
| B5 | 0.8526 | 1.0324 | -3216.1282 | -3321.2865 |
| B6 | 0.8587 | 1.0278 | -755.7922 | -861.0841 |

Table S3 Computed NBO parameters and absolute electronic energies at the B97-D3(BJ)/genecp level with the SMD implicit solvation model for water.

| Compound | WBI (C_ipso_–B) | WBI (C_ipso_–At) | Absolute electronic energy of pre-labeling precursor | Absolute electronic energy of post-labeling product |
| --- | --- | --- | --- | --- |
| B1 | 0.9148 | 1.0386 | -871.774 | -938.6316 |
| B2 | 0.9099 | 0.9792 | -697.3752 | -549.5791 |
| B3 | 0.9116 | 0.9965 | -962.8416 | -815.0381 |
| B4 | 0.8283 | 0.9835 | -940.4001 | -1045.9396 |
| B5 | 0.8411 | 0.9868 | -3216.954 | -3323.1513 |
| B6 | 0.8602 | 0.9798 | -755.2164 | -861.7691 |

Table S4 Computed NBO parameters and absolute electronic energies at the B97-D3(BJ)/genecp level with the IEFPCM implicit solvation model for water.

| Compound | WBI (C_ipso_–B) | WBI (C_ipso_–At) | Absolute electronic energy of pre-labeling precursor | Absolute electronic energy of post-labeling product |
| --- | --- | --- | --- | --- |
| B1 | 0.9174 | 0.7704 | -871.7754 | -938.6212 |
| B2 | 0.9074 | 0.9813 | -697.3741 | -549.5778 |
| B3 | 0.9047 | 0.9976 | -962.8115 | -815.0391 |
| B4 | 0.8217 | 0.9818 | -940.4009 | -1045.9401 |
| B5 | 0.8515 | 0.9732 | -3216.9553 | -3323.149 |
| B6 | 0.8623 | 0.9771 | -755.2604 | -861.7658 |

**Cartesian coordinates for all optimized aryl boronic ester precursors and their corresponding ^211^At-labeled derivatives are provided to ensure reproducibility and facilitate future comparative modeling. Identical starting geometries were used consistently across the primary calculations and all benchmarking protocols.**

*2-(3,4-dimethoxyphenyl)-4,4,5,5-tetramethyl-1,3,2-dioxaborolane*

C	-0.87434797	-0.78603508	0.00068200

C	0.52081203	-0.78603508	0.00068200

C	1.21835003	0.42171592	0.00068200

C	0.52069603	1.63022492	-0.00051700

C	-0.87412897	1.63014692	-0.00099600

C	-1.57172997	0.42194092	0.00000000

H	2.31803003	0.42179592	0.00131600

H	1.07089603	2.58236792	-0.00057600

H	-2.67133397	0.42212392	-0.00018000

B	-1.69949131	3.05887886	-0.00242581

O	-2.08805539	3.72499388	1.33058160

O	-2.08126542	3.72624700	-1.33676771

C	-2.78557817	4.94344351	-1.07739110

C	-2.79023995	4.94289816	1.06876868

C	-3.16719378	5.61012852	-2.41211978

H	-3.27535965	4.85997059	-3.16740994

H	-4.09191422	6.13591621	-2.29662338

H	-2.39945583	6.29771479	-2.69970203

C	-3.17795663	5.60872935	2.40216457

H	-2.29555553	5.79315771	2.97857976

H	-3.67631414	6.53516476	2.20657005

H	-3.83138770	4.96048830	2.94779392

C	-1.99366083	6.52183771	-0.68036734

H	-1.63700640	5.51302770	-0.68036734

H	-1.63698799	7.02623590	0.19328417

H	-1.63698799	7.02623590	-1.55401884

C	-1.13645309	6.02480916	2.30812712

H	-0.77979866	5.01599916	2.30812712

H	-0.77978025	6.52920735	3.18177862

H	-0.77978025	6.52920735	1.43447561

O	1.23539635	-2.02469013	0.00239204

C	1.35976979	-2.50587272	-1.33846387

H	1.95575469	-3.39450287	-1.34466500

H	0.38851364	-2.72507581	-1.73026930

H	1.82810365	-1.75808503	-1.94375467

O	-1.58936465	-2.02444158	0.00138118

C	-2.11814993	-2.26959400	1.30720904

H	-3.07542925	-1.80030962	1.39825004

H	-2.22198064	-3.32383979	1.45782447

H	-1.45270419	-1.86806822	2.04264023

*1-[^211^At]astato-3,4-dimethoxybenzene*

C	-0.87434797	-0.78603508	0.00068200

C	0.52081203	-0.78603508	0.00068200

C	1.21835003	0.42171592	0.00068200

C	0.52069603	1.63022492	-0.00051700

C	-0.87412897	1.63014692	-0.00099600

C	-1.57172997	0.42194092	0.00000000

H	2.31803003	0.42179592	0.00131600

H	1.07089603	2.58236792	-0.00057600

H	-2.67133397	0.42212392	-0.00018000

O	1.23539635	-2.02469013	0.00239204

C	1.35976979	-2.50587272	-1.33846387

H	1.95575469	-3.39450287	-1.34466500

H	0.38851364	-2.72507581	-1.73026930

H	1.82810365	-1.75808503	-1.94375467

O	-1.58936465	-2.02444158	0.00138118

C	-2.11814993	-2.26959400	1.30720904

H	-3.07542925	-1.80030962	1.39825004

H	-2.22198064	-3.32383979	1.45782447

H	-1.45270419	-1.86806822	2.04264022

At	-2.01963186	3.61305368	-0.00298040

*4-(4,4,5,5-tetramethyl-1,3,2-dioxaborolan-2-yl)aniline*

C	-0.87434797	-0.78603508	0.00068200

C	0.52081203	-0.78603508	0.00068200

C	1.21835003	0.42171592	0.00068200

C	0.52069603	1.63022492	-0.00051700

C	-0.87412897	1.63014692	-0.00099600

C	-1.57172997	0.42194092	0.00000000

H	2.31803003	0.42179592	0.00131600

H	1.07089603	2.58236792	-0.00057600

H	-2.67133397	0.42212392	-0.00018000

B	-1.69949131	3.05887886	-0.00242581

O	-2.08805539	3.72499388	1.33058160

O	-2.08126542	3.72624700	-1.33676771

C	-2.78557817	4.94344351	-1.07739110

C	-2.79023995	4.94289816	1.06876868

C	-3.16719378	5.61012852	-2.41211978

H	-3.27535965	4.85997059	-3.16740994

H	-4.09191422	6.13591621	-2.29662338

H	-2.39945583	6.29771479	-2.69970203

C	-3.17795663	5.60872935	2.40216457

H	-2.29555553	5.79315771	2.97857976

H	-3.67631414	6.53516476	2.20657005

H	-3.83138770	4.96048830	2.94779392

C	-1.99366083	6.52183771	-0.68036734

H	-1.63700640	5.51302770	-0.68036734

H	-1.63698799	7.02623590	0.19328417

H	-1.63698799	7.02623590	-1.55401884

C	-1.13645309	6.02480916	2.30812712

H	-0.77979866	5.01599916	2.30812712

H	-0.77978025	6.52920735	3.18177862

H	-0.77978025	6.52920735	1.43447561

H	-1.40936045	-1.71267491	0.00120516

N	1.25538473	-2.05933782	0.00243987

H	0.97735598	-2.60346636	0.79403582

H	1.05100583	-2.56322898	-0.83679965

*4-[^211^At]astatobenzenamine*

C	-0.87434797	-0.78603508	0.00068200

C	0.52081203	-0.78603508	0.00068200

C	1.21835003	0.42171592	0.00068200

C	0.52069603	1.63022492	-0.00051700

C	-0.87412897	1.63014692	-0.00099600

C	-1.57172997	0.42194092	0.00000000

H	2.31803003	0.42179592	0.00131600

H	1.07089603	2.58236792	-0.00057600

H	-2.67133397	0.42212392	-0.00018000

At	-2.01963186	3.61305368	-0.00298040

H	-1.40936045	-1.71267491	0.00120516

N	1.25538473	-2.05933782	0.00243987

H	1.82855404	-2.11462884	-0.81512960

H	1.83200688	-2.11038246	0.81785470

*2-(4,4,5,5-tetramethyl-1,3,2-dioxaborolan-2-yl)thiophene*

| C | -3.79816 | -3.79816 | -3.79816 |
| --- | --- | --- | --- |
| C | -3.76779 | -3.76779 | -3.76779 |
| C | -2.43975 | -2.43975 | -2.43975 |
| C | -1.46402 | -1.46402 | -1.46402 |
| S | -2.21246 | -2.21246 | -2.21246 |
| H | -4.67348 | -4.67348 | -4.67348 |
| H | -4.66487 | -4.66487 | -4.66487 |
| H | -2.20057 | -2.20057 | -2.20057 |
| B | 0.07131 | 0.07131 | 0.07131 |
| O | 0.63273 | 0.63273 | 0.63273 |
| O | 0.85686 | 0.85686 | 0.85686 |
| C | 2.31541 | 2.31541 | 2.31541 |
| C | 0.44781 | 0.44781 | 0.44781 |
| C | 2.58314 | 2.58314 | 2.58314 |
| H | 3.66558 | 3.66558 | 3.66558 |
| H | 2.14083 | 2.14083 | 2.14083 |
| H | 2.14685 | 2.14685 | 2.14685 |
| C | 1.13998 | 1.13998 | 1.13998 |
| H | 0.47617 | 0.47617 | 0.47617 |
| H | 1.53225 | 1.53225 | 1.53225 |
| H | 1.97616 | 1.97616 | 1.97616 |
| C | 2.87429 | 2.87429 | 2.87429 |
| H | 2.68604 | 2.68604 | 2.68604 |
| H | 3.96156 | 3.96156 | 3.96156 |
| H | 2.41933 | 2.41933 | 2.41933 |
| C | 2.87403 | 2.87403 | 2.87403 |
| H | 2.39289 | 2.39289 | 2.39289 |
| H | 3.95524 | 3.95524 | 3.95524 |
| H | 2.72224 | 2.72224 | 2.72224 |

*4-[^211^At]astatothiphene*

| C | -3.46453 | -0.21282 | -0.000174 |
| --- | --- | --- | --- |
| C | -3.15242 | 1.12307 | 0.000088 |
| C | -1.74269 | 1.37085 | -0.000001 |
| C | -1.01487 | 0.20562 | -0.000024 |
| S | -2.03823 | -1.19993 | 0.000070 |
| H | -4.44998 | -0.67884 | -0.000281 |
| H | -3.90388 | 1.91447 | 0.000158 |
| H | -1.29424 | 2.36459 | 0.000033 |
| At | 1.15890 | 0.00798 | -0.000004 |

5*,5-Dimethyl-2-(2-(trifluoromethyl)phenyl)-1,3,2-dioxaborinane*

| C | -3.39001 | 0.48996 | -0.11494 |
| --- | --- | --- | --- |
| C | -3.59307 | 1.87001 | -0.17557 |
| C | -2.49858 | 2.73535 | -0.11359 |
| C | -1.20600 | 2.21737 | 0.00455 |
| C | -0.97062 | 0.83289 | 0.04621 |
| C | -2.09154 | -0.02061 | -0.00813 |
| H | -4.24310 | -0.18933 | -0.14233 |
| H | -4.60717 | 2.26613 | -0.26152 |
| H | -2.65202 | 3.81637 | -0.15188 |
| H | -0.35685 | 2.90291 | 0.06020 |
| B | 0.52017 | 0.32881 | 0.14502 |
| O | 1.28760 | 0.81259 | 1.16183 |
| O | 1.00390 | -0.50753 | -0.81492 |
| C | 2.35895 | -0.96854 | -0.73699 |
| H | 2.68914 | -1.18935 | -1.76312 |
| H | 2.37079 | -1.90984 | -0.16261 |
| C | 2.66245 | 0.41858 | 1.27620 |
| H | 2.71625 | -0.44789 | 1.95555 |
| H | 3.20433 | 1.25382 | 1.74498 |
| C | 3.28057 | 0.06478 | -0.08063 |
| C | 4.65989 | -0.55966 | 0.14244 |
| H | 5.33272 | 0.15450 | 0.64298 |
| H | 4.59561 | -1.46475 | 0.76701 |
| H | 5.11890 | -0.83905 | -0.81907 |
| C | 3.40070 | 1.31695 | -0.96161 |
| H | 3.82831 | 1.05752 | -1.94280 |
| H | 2.42642 | 1.79799 | -1.13733 |
| H | 4.06017 | 2.05939 | -0.48550 |
| C | -1.90648 | -1.51419 | 0.05588 |
| F | -1.57295 | -2.05058 | -1.13959 |
| F | -0.92261 | -1.86701 | 0.92137 |
| F | -3.02154 | -2.15467 | 0.46180 |

*4-[^211^At]astato-2-(trifluoromethyl)benzene*

| C | 2.97884 | 0.56897 | 0.00003 |
| --- | --- | --- | --- |
| C | 3.13078 | 1.95538 | 0.00004 |
| C | 2.00156 | 2.77436 | -0.00001 |
| C | 0.72303 | 2.20895 | -0.00005 |
| C | 0.57178 | 0.81969 | -0.00008 |
| C | 1.70426 | -0.01514 | -0.00006 |
| H | 3.85865 | -0.07459 | 0.00007 |
| H | 4.13173 | 2.39077 | 0.00009 |
| H | 2.10583 | 3.86162 | 0.00001 |
| H | -0.15433 | 2.85692 | -0.00005 |
| C | 1.60548 | -1.52181 | -0.00001 |
| F | 0.94723 | -1.99031 | 1.08368 |
| F | 0.94839 | -1.99048 | -1.08433 |
| F | 2.81521 | -2.11091 | 0.00067 |
| At | -1.51334 | 0.05939 | 0.00001 |

*2-(2-(Bromomethyl)phenyl)-5,5-dimethyl-1,3,2-dioxaborinane*

| C | -1.12955 | -1.12955 | -1.12955 |
| --- | --- | --- | --- |
| C | 0.96787 | 0.96787 | 0.96787 |
| C | 0.56721 | 0.56721 | 0.56721 |
| C | -0.63670 | -0.63670 | -0.63670 |
| C | -1.88811 | -1.88811 | -1.88811 |
| C | -2.13844 | -2.13844 | -2.13844 |
| H | 1.31094 | 1.31094 | 1.31094 |
| H | -0.68439 | -0.68439 | -0.68439 |
| H | -2.77499 | -2.77499 | -2.77499 |
| H | -3.19424 | -3.19424 | -3.19424 |
| B | 1.18121 | 1.18121 | 1.18121 |
| O | 1.66911 | 1.66911 | 1.66911 |
| O | 1.66867 | 1.66867 | 1.66867 |
| C | 2.15556 | 2.15556 | 2.15556 |
| H | 1.31723 | 1.31723 | 1.31723 |
| H | 2.73051 | 2.73051 | 2.73051 |
| C | 2.15533 | 2.15533 | 2.15533 |
| H | 1.31679 | 1.31679 | 1.31679 |
| H | 2.73004 | 2.73004 | 2.73004 |
| C | 3.03619 | 3.03619 | 3.03619 |
| C | 3.47215 | 3.47215 | 3.47215 |
| H | 4.07937 | 4.07937 | 4.07937 |
| H | 2.60277 | 2.60277 | 2.60277 |
| H | 4.07990 | 4.07990 | 4.07990 |
| C | 4.25098 | 4.25098 | 4.25098 |
| H | 3.92788 | 3.92788 | 3.92788 |
| H | 4.87608 | 4.87608 | 4.87608 |
| H | 4.87551 | 4.87551 | 4.87551 |
| C | -1.30967 | -1.30967 | -1.30967 |
| H | -0.88074 | -0.88074 | -0.88074 |
| H | -0.88048 | -0.88048 | -0.88048 |
| Br | -3.28735 | -3.28735 | -3.28735 |

*4-[^211^At]astato-2(bromomethyl)benzene*

| C | -1.08031 | 0.73726 | 0.000234 |
| --- | --- | --- | --- |
| C | 0.28916 | 1.06749 | -0.000093 |
| C | 0.72699 | 2.39359 | -0.000308 |
| C | -0.21047 | 3.43050 | -0.000187 |
| C | -1.57270 | 3.12993 | 0.000158 |
| C | -1.99615 | 1.79894 | 0.000360 |
| H | 1.79371 | 2.62227 | -0.000579 |
| H | 0.13204 | 4.46767 | -0.000386 |
| H | -2.31478 | 3.93115 | 0.000272 |
| H | -3.06359 | 1.57574 | 0.000581 |
| C | -1.48564 | -0.71594 | 0.000511 |
| H | -1.10460 | -1.23391 | -0.888361 |
| H | -1.10531 | -1.23336 | 0.890019 |
| Br | -3.41930 | -1.07165 | -0.000160 |
| At | 1.85074 | -0.51379 | 0.000000 |

*1-(4-(5,5-Dimethyl-1,3,2-dioxaborinan-2-yl)phenyl)ethan-1-one*

| C | 3.23369 | 3.23369 | 3.23369 |
| --- | --- | --- | --- |
| C | 2.50427 | 2.50427 | 2.50427 |
| C | 1.11721 | 1.11721 | 1.11721 |
| C | 0.40991 | 0.40991 | 0.40991 |
| C | 1.14998 | 1.14998 | 1.14998 |
| C | 2.54035 | 2.54035 | 2.54035 |
| H | 3.04526 | 3.04526 | 3.04526 |
| H | 0.56736 | 0.56736 | 0.56736 |
| H | 0.62741 | 0.62741 | 0.62741 |
| H | 3.08165 | 3.08165 | 3.08165 |
| B | -1.15772 | -1.15772 | -1.15772 |
| O | -1.82838 | -1.82838 | -1.82838 |
| O | -1.79705 | -1.79705 | -1.79705 |
| C | -3.25781 | -3.25781 | -3.25781 |
| H | -3.60755 | -3.60755 | -3.60755 |
| H | -3.53942 | -3.53942 | -3.53942 |
| C | -3.22548 | -3.22548 | -3.22548 |
| H | -3.50560 | -3.50560 | -3.50560 |
| H | -3.55108 | -3.55108 | -3.55108 |
| C | -3.89289 | -3.89289 | -3.89289 |
| C | -5.38967 | -5.38967 | -5.38967 |
| H | -5.88892 | -5.88892 | -5.88892 |
| H | -5.56989 | -5.56989 | -5.56989 |
| H | -5.86561 | -5.86561 | -5.86561 |
| C | -3.67534 | -3.67534 | -3.67534 |
| H | -4.12367 | -4.12367 | -4.12367 |
| H | -2.60814 | -2.60814 | -2.60814 |
| H | -4.14653 | -4.14653 | -4.14653 |
| C | 4.72350 | 4.72350 | 4.72350 |
| C | 5.50190 | 5.50190 | 5.50190 |
| H | 6.57181 | 6.57181 | 6.57181 |
| H | 5.17214 | 5.17214 | 5.17214 |
| H | 5.32929 | 5.32929 | 5.32929 |
| O | 5.29573 | 5.29573 | 5.29573 |

*4-[^211^At]astatoacetophenone*

| C | -2.93889 | 0.07067 | -0.000198 |
| --- | --- | --- | --- |
| C | -2.20340 | 1.26829 | -0.000338 |
| C | -0.81054 | 1.25414 | -0.000371 |
| C | -0.14473 | 0.02261 | -0.000337 |
| C | -0.85240 | -1.18278 | -0.000471 |
| C | -2.24833 | -1.15254 | -0.000420 |
| H | -2.74001 | 2.21840 | -0.000370 |
| H | -0.25451 | 2.19257 | -0.000478 |
| H | -0.33012 | -2.14023 | -0.000611 |
| H | -2.79092 | -2.09857 | -0.000711 |
| C | -4.43313 | 0.14557 | 0.000118 |
| C | -5.22138 | -1.13709 | 0.000419 |
| H | -6.29409 | -0.90622 | 0.000375 |
| H | -4.97272 | -1.74294 | -0.885301 |
| H | -4.97273 | -1.74250 | 0.886464 |
| O | -4.99819 | 1.23358 | 0.000322 |
| At | 2.06420 | -0.01626 | 0.000090 |

**References:**

(1) Becke, A. D. Density-functional thermochemistry. III. The role of exact exchange. *J. Chem. Phys.* **1993**, *98* (7), 5648-5652. DOI: <https://doi.org/10.1063/1.464913>.

(2) Grimme, S.; Ehrlich, S.; Goerigk, L. Effect of the damping function in dispersion corrected density functional theory. *J Comput Chem* **2011**, *32* (7), 1456-1465. DOI: <https://doi.org/10.1002/jcc.21759>.

(3) Weigend, F.; Ahlrichs, R. Balanced basis sets of split valence, triple zeta valence and quadruple zeta valence quality for H to Rn: Design and assessment of accuracy. *Phys Chem Chem Phys* **2005**, *7* (18), 3297-3305. DOI: <https://doi.org/10.1039/B508541A>.

(4) Marenich, A. V.; Cramer, C. J.; Truhlar, D. G. Universal solvation model based on solute electron density and on a continuum model of the solvent defined by the bulk dielectric constant and atomic surface tensions. *J Chem Phys* **2009**, *113* (18), 6378-6396. DOI: <https://doi.org/10.1021/jp810292n>.

(5) Shaffer, A. A.; Wierschke, S. G. Comparison of computational methods applied to oxazole, thiazole, and other heterocyclic compounds. *Journal of Computational Chemistry* **1993**, *14* (1), 75-88. DOI: <https://doi.org/10.1002/jcc.540140112>.

(6) Mayer, I. Bond order and valence indices: a personal account. *J. Comput. Chem.* **2007**, *28* (1), 204-221. DOI: <https://doi.org/10.1002/jcc.20494>.

(7) Pomogaeva, A. V.; Timoshkin, A. Y. Stability and Electronic Structure of Donor-Acceptor Stabilized Group 13/15 Oligomers. *J Phys Chem A* **2021**, *125* (16), 3415-3424. DOI: <https://doi.org/10.1021/acs.jpca.1c02258>.

(8) Cances, E.; Mennucci, B.; Tomasi, J. A new integral equation formalism for the polarizable continuum model: Theoretical background and applications to isotropic and anisotropic dielectrics. *Journal of Chemical Physics* **1997**, *107* (8), 3032-3041. DOI: <https://doi.org/10.1063/1.474659>.

(9) Bitter, T.; Ruedenberg, K.; Schwarz, W. H. Toward a physical understanding of electron-sharing two-center bonds. I. General aspects. *J Comput Chem* **2006**, *28* (1), 411-422. DOI: <https://doi.org/10.1002/jcc.20531>.

(10) Champion, J.; Alliot, C.; Renault, E.; Mokili, B. M.; Cherel, M.; Galland, N.; Montavon, G. Astatine standard redox potentials and speciation in acidic medium. *J Phys Chem A* **2010**, *114* (1), 576-582. DOI: <https://doi.org/10.1021/jp9077008>.
